# Case Report: Pelvic splenosis confused with malignancy—a reminder for differential diagnosis

**DOI:** 10.3389/fonc.2026.1746336

**Published:** 2026-03-30

**Authors:** Bo Cheng, Xiwen Zhang, Meiqi Fu, Peicong Gao, Jiayi Feng, Fei Wu

**Affiliations:** Department of Obstetrics and Gynecology, The Second Hospital of Jilin University, Changchun, Jilin, China

**Keywords:** case report, misdiagnosis, multidisciplinary consultation, pelvic mass, splenosis

## Abstract

**Background:**

Splenosis is a rare, benign disorder that arises after splenic trauma or splenectomy. Because its imaging features are nonspecific and often mimic those of malignant tumors, it is frequently misdiagnosed as malignant. The condition often presents without specific symptoms or elevated tumor markers, further complicating the diagnostic process.

**Case presentation:**

A 47-year-old woman with a prior history of splenectomy 20 years earlier after trauma presented with a progressively enlarging pelvic mass. Serial imaging modalities, including magnetic resonance imaging, contrast-enhanced ultrasound, and computed tomography, indicated a potential gastrointestinal malignancy, although all tumor markers remained within normal limits. Image-guided percutaneous biopsy was considered but deemed unsuitable due to the complex anatomical location of the dominant mass in the rectouterine pouch, where a safe needle trajectory avoiding bowel and major vessels could not be guaranteed. Exploratory surgery was performed, and intraoperative frozen-section analysis of an approximately 5-cm purple-brown nodule confirmed the diagnosis of splenosis. Histopathological examination with immunohistochemical staining (CD8+, CD68+, CD117-, DOG1-) confirmed the diagnosis of splenosis and excluded malignancies such as gastrointestinal stromal tumor (GIST) and carcinoma. Smaller implants located in the omentum and mesentery were preserved. The patient’s postoperative course was uneventful, and she expressed both relief and satisfaction with the benign diagnosis as well as the avoidance of unnecessary extensive resection.

**Conclusion:**

This case highlights the necessity of considering splenosis in the differential diagnosis of pelvic masses, especially in patients with a history of splenic trauma or splenectomy. A comprehensive evaluation that incorporates clinical history, advanced imaging modalities—including magnetic resonance imaging and ^99mTc-labeled heat-damaged red blood cell scintigraphy—as well as multidisciplinary consultation, can enhance diagnostic accuracy and help prevent overtreatment. Current evidence indicates that asymptomatic splenosis does not necessitate surgical intervention, and precise preoperative recognition is crucial for optimizing patient management.

## 1 Introduction

Splenosis is an autotransplantation phenomenon that most commonly arises following splenic trauma or splenectomy (1). Owing to its close resemblance to malignant tumors on imaging, splenosis is frequently misdiagnosed (2, 3). The abdominal, pelvic, and thoracic cavities are the most frequent sites of involvement, and rare ectopic locations have also been documented (4, 5). The diagnostic challenge is compounded by the fact that splenosis typically presents without specific clinical symptoms and is often associated with normal tumor marker levels, further blurring the distinction from malignant conditions. Because of its nonspecific imaging features, definitive diagnosis typically requires histopathological confirmation, which presents considerable challenges in clinical practice. We report a case of splenosis misdiagnosed as a pelvic malignancy and discuss the key aspects of differential diagnosis and treatment strategies to improve clinicians’ awareness, recognition, and management of this rare condition.

## 2 Case presentation

**Figure 1:**
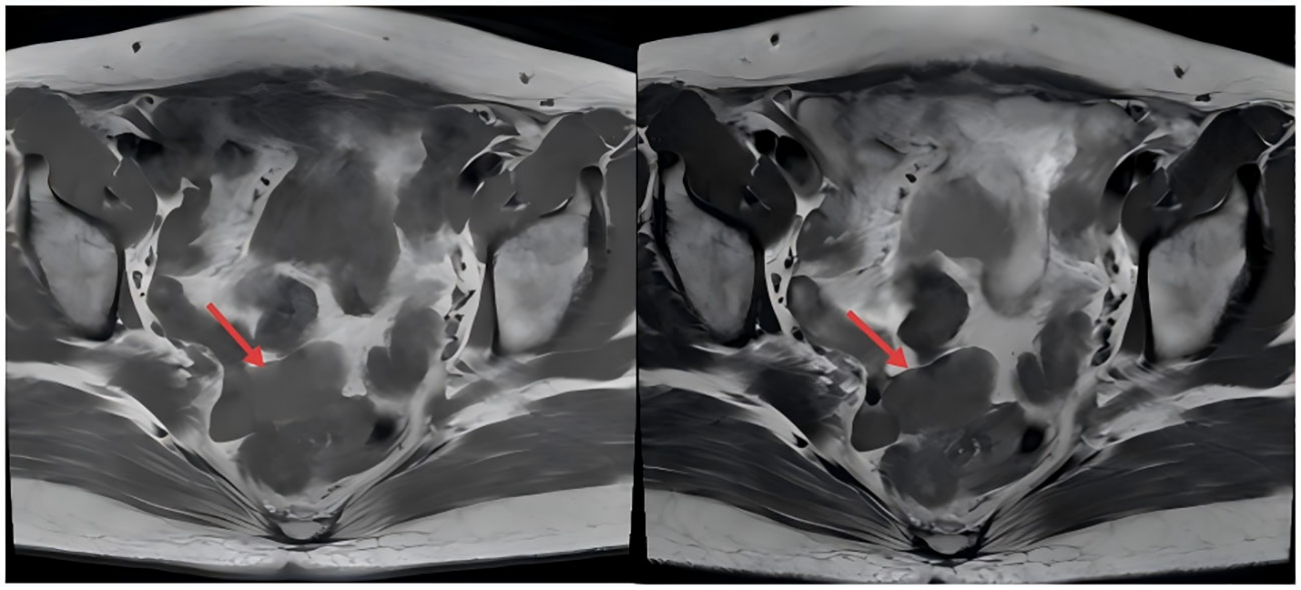
Magnetic Resonance Imaging (MRI) T2-weighted (2023). Red arrows indicate the nodular lesions in the rectouterine pouch.

**Figure 2:**
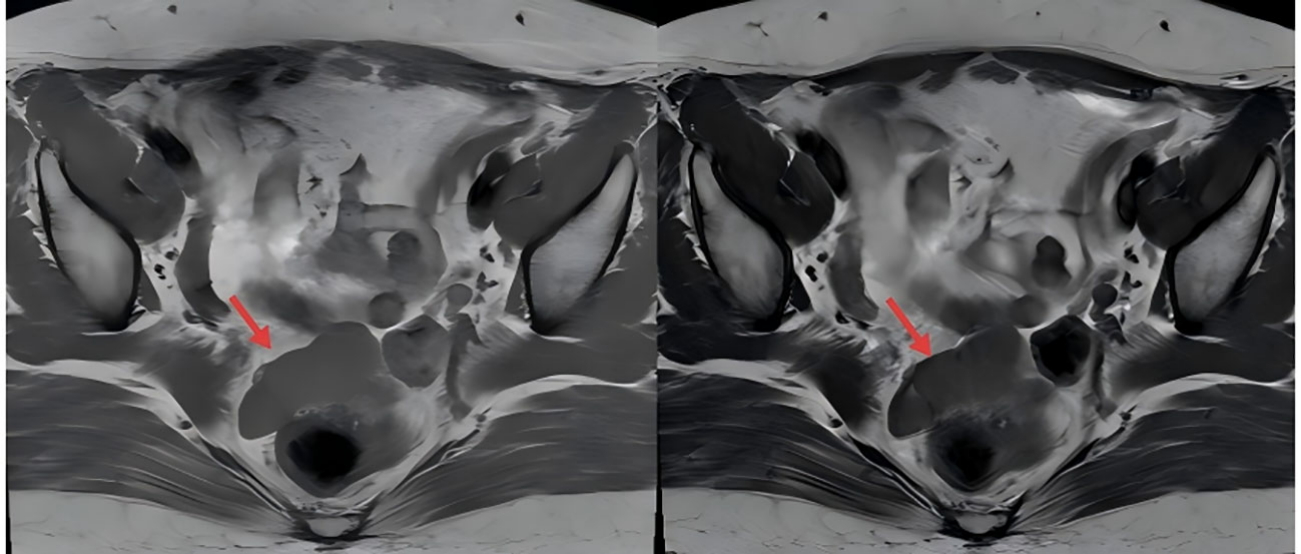
Magnetic Resonance Imaging (MRI) T2-weighted (2024). Red arrows show the same lesions, demonstrating no significant interval change.

A 47-year-old woman was incidentally found to have a pelvic mass on a gynecological ultrasound during a routine health examination 4 years earlier. Serial follow-up examinations demonstrated progressive enlargement of the mass. Pelvic MRI performed 1 year earlier revealed multiple nodular lesions in the rectouterine pouch, which appeared isointense on T1-weighted images and slightly hypointense on T2-weighted images. (**Figure 1**, red arrows) The most extensive lesion measured approximately 42 × 24 × 18 mm. The lesions showed mild enhancement after contrast administration and were poorly delineated from the posterior aspect of the cervix. No apparent abnormalities were detected in the bladder or rectum, and both adnexa appeared unremarkable. Additionally, a nodular enhancing lesion measuring approximately 6 × 5 mm was identified in the anterior uterine wall, although it was poorly visualized on non-contrast images. Lymph nodes were observed adjacent to the bilateral iliac vessels, with the largest located on the left side, measuring approximately 10 mm in short-axis diameter. The nodules in the rectouterine pouch were considered likely benign, and lymph nodes were observed adjacent to the iliac vessels. A repeat MRI performed before admission showed no significant changes compared with previous imaging (**Figure 2**, red arrows).

The patient had no family history of malignancy but she had a history of splenectomy following a traffic accident 20 years prior to the discovery of the pelvic mass. On gynecological examination, a firm nodular mass about 5.0 × 4.0 × 5.0 cm was palpated behind the uterus. It was moderately mobile, with relatively clear margins, and there was no tenderness or rebound pain. Serum tumor markers were all within the normal range: CA125, 22.2 U/mL; CA19-9, <2.00 U/mL; HE4, 36.9 pmol/L; AFP, 1.70 ng/mL; CEA, 2.32 ng/mL; CA724, 4.36 U/mL; CA50, 0.50 U/mL; CA242, 0.50 U/mL; and SF, 69.19 ng/mL.

Gynecological ultrasound revealed two solid hypoechoic tubular masses located posterior to the cervix, measuring 5.9 × 2.0 cm and 4.2 × 1.5 cm, with well-defined margins. Color Doppler flow imaging (CDFI) demonstrated sparse internal blood flow within the lesions. Contrast-enhanced ultrasound was performed after intravenous bolus injection of 1.8 mL sulfur hexafluoride microbubble suspension through a 22-G catheter in the cubital vein. During the early enhancement phase, the lesions exhibited rapid, diffuse perfusion earlier than the myometrium and demonstrated homogeneous hyperenhancement at peak intensity. In the late phase, enhancement persisted without washout, showing sustained hyperenhancement. These findings suggested a possible intestinal origin, for which further computed tomography (CT) evaluation was recommended.

**Figure 3:**
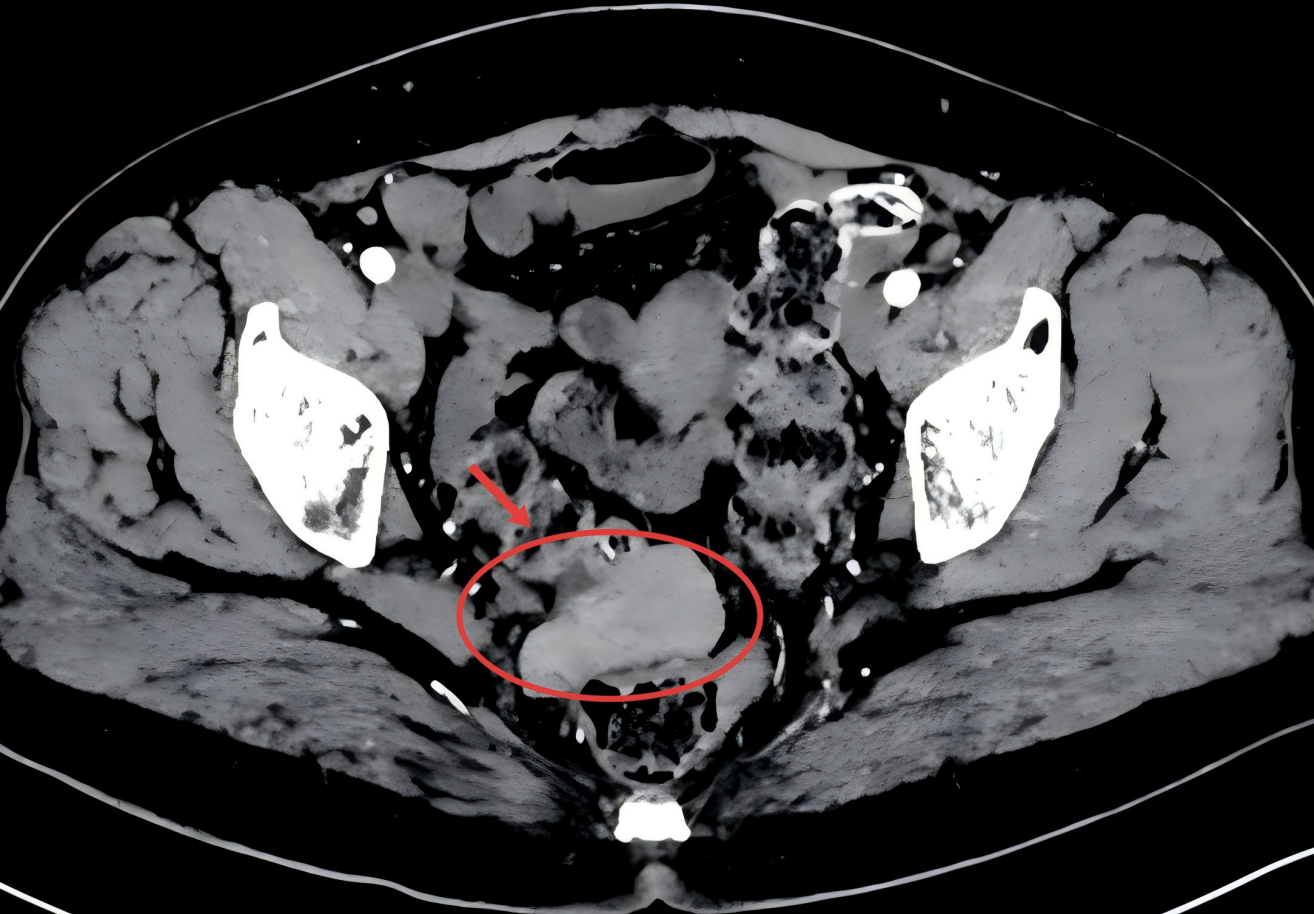
Contrast-enhanced CT of the abdomen. Red arrows indicate the nodular lesions in the rectouterine pouch.

Contrast-enhanced abdominal CT revealed multiple nodular masses involving the rectouterine pouch, mesentery, and omentum (**Figure 3**, red arrows), for which histopathological confirmation was recommended. Multiple lymph nodes were also identified in the retroperitoneum and along the bilateral iliac vessels, with partial enlargement of the left iliac nodes. Additional findings included a cystic lesion in the right adnexa, pelvic effusion, postsplenectomy, and a left renal cyst. Based on the available imaging and laboratory findings, the possibility of a pelvic malignancy of intestinal origin could not be excluded.

**Figure 4:**
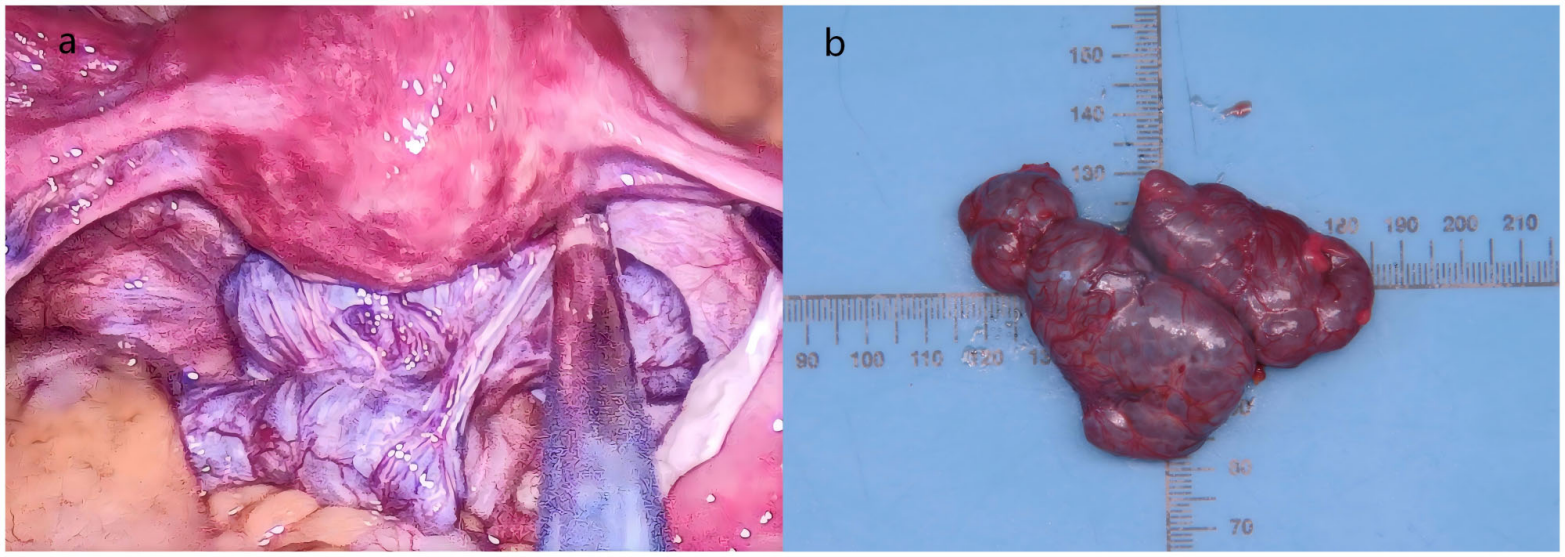
Intraoperative exploration image. **(a)** A smooth, purple-brown mass (approximately 5 × 4 cm) is visible posterior to the uterus, adherent to the rectum. **(b)** Close-up view of the resected nodule, demonstrating the characteristic purple-brown color and smooth surface.

To establish a definitive diagnosis, an exploratory laparotomy was performed on January 13, 2025. Although imaging-guided percutaneous biopsy was considered, it was ultimately deemed unsuitable due to the complex anatomical location of the dominant mass in the rectouterine pouch, where a safe needle trajectory avoiding bowel and major vessels could not be guaranteed. The risk of complications such as bleeding, infection, or tumor seeding, coupled with the potential for insufficient sampling to differentiate between entities like gastrointestinal stromal tumor (GIST) and splenosis, led the multidisciplinary team to favor a diagnostic surgical approach that could also be therapeutic if malignancy were confirmed. Intraoperatively, the uterus, bilateral fallopian tubes, and ovaries appeared grossly unremarkable. A smooth purple-brown mass (≈5 × 4 cm) lay behind the uterus, adherent to the intestinal tract. Several purple-brown nodules, ranging from 1.0 to 2.0 cm in diameter, were observed on the omental and mesenteric surfaces (**Figure 4a**). Because an intestinal origin was suspected, the gastrointestinal surgery team was invited for intraoperative consultation, and a gastrointestinal stromal tumor (GIST) was considered. The posterior pelvic mass was removed with an ultrasonic scalpel and sent for frozen section (**Figure 4b**). Pathology confirmed splenosis. The remaining implants on the greater omentum and mesentery were preserved. The patient recovered without issues and was reassured by the benign diagnosis and the avoidance of an extensive resection.

## 3 Discussion

Splenosis is a benign condition caused by the autotransplantation of splenic tissue, most commonly occurring after splenic trauma or splenectomy (6). However, because of its striking radiological resemblance to malignant tumors, establishing a definitive diagnosis is often highly challenging. In the present case, pelvic splenosis was initially suspected to represent a malignancy, underscoring the considerable difficulty of preoperative differential diagnosis. Although several clinical and radiological features suggested a benign process—for example, normal tumor marker levels and the absence of symptoms—contrast-enhanced ultrasound (CEUS) and CT could not definitively exclude malignancy, further emphasizing the diagnostic dilemma.

The diagnosis of splenosis requires careful integration of the patient’s clinical history, imaging findings, and histopathological confirmation. In patients with a history of splenic trauma or splenectomy, splenosis should be strongly considered when nodules are detected in the peritoneal or pelvic cavities (7, 8). Technetium-99m–labeled heat-damaged red blood cell (RBC) scintigraphy, which highlights explicitly functional splenic tissue, is considered the gold standard for noninvasive diagnosis (9, 10). Unfortunately, this technique is not widely available in most hospitals, including our institution, and was therefore not performed preoperatively in this case. Because transplanted splenic tissue often exhibits irregular morphology, the assessment of lesion size may vary depending on the ultrasound operator and imaging plane. In this case, although ultrasound suggested enlargement of the pelvic mass, MRI showed no significant interval change, indicating that MRI may be more reliable than ultrasound for differentiating splenosis from pelvic tumors. Furthermore, superparamagnetic iron oxide (SPIO)–enhanced MRI, which exploits the unique phagocytic properties of splenic tissue, can further assist in distinguishing splenosis from malignancy (11).

The option of image-guided percutaneous biopsy was carefully evaluated but ultimately not pursued. The dominant pelvic lesion’s location within the rectouterine pouch, intimately associated with the intestinal tract and adjacent to pelvic vessels, posed a significant risk for procedure-related complications and did not offer a safe puncture path. Additionally, the limited sample size from a core needle biopsy might have been insufficient for a definitive diagnosis, particularly to confidently exclude a hypocellular or stroma-rich malignancy. Given these technical limitations and the potential for sampling error, proceeding directly to diagnostic laparoscopy with the capacity for both frozen section analysis and definitive resection was considered the most appropriate and decisive approach.

**Figure 5:**
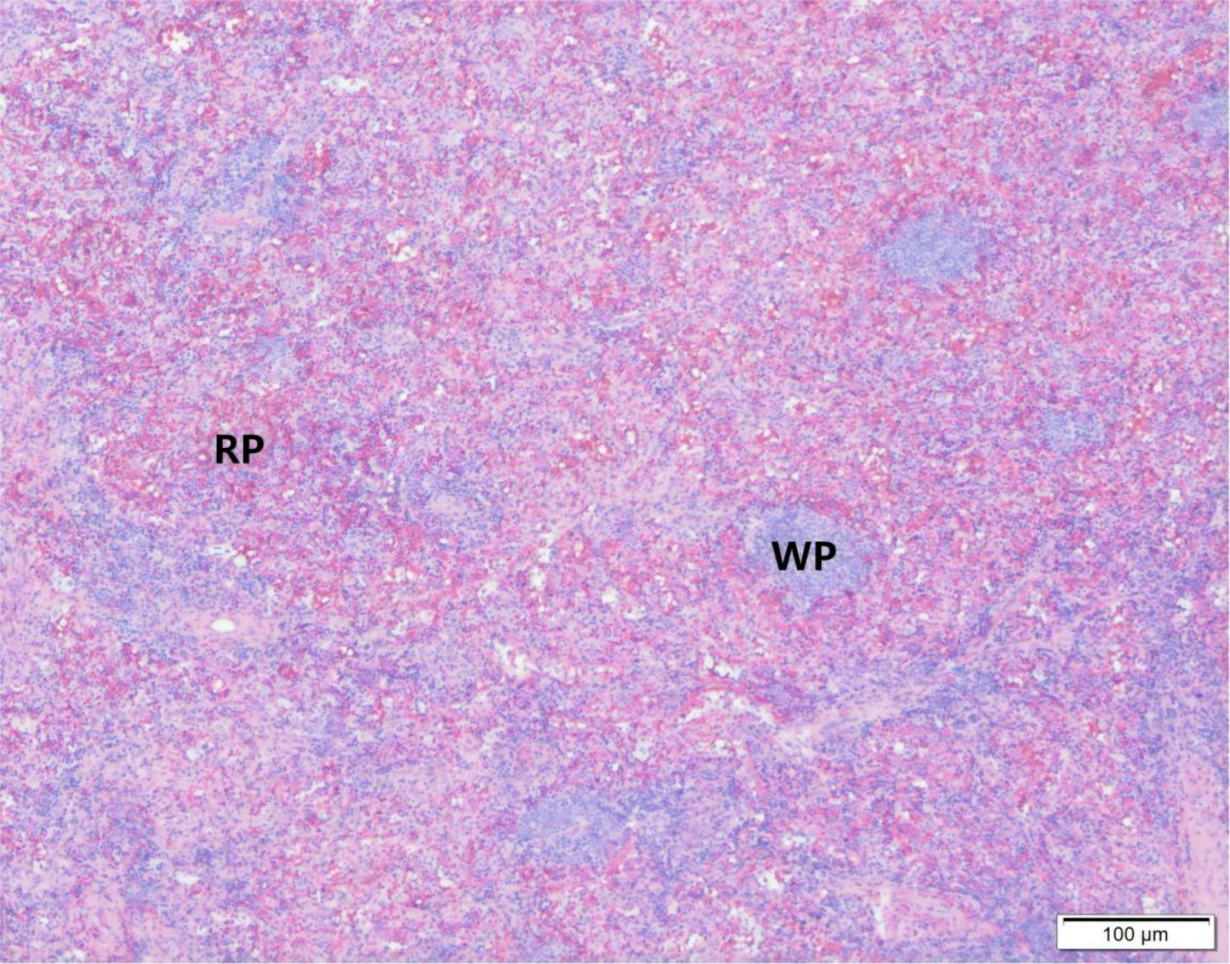
Histological section of splenic nodule stained with hematoxylin and eosin (H&E). The image demonstrates normal splenic architecture with distinct white pulp (WP) lymphoid follicles appearing as basophilic (blue-purple) nodules scattered within the eosinophilic (pink-red) red pulp (RP). No cytological atypia or neoplastic infiltration is identified in this field. Scale bar = 100 μm.

**Figure 6:**
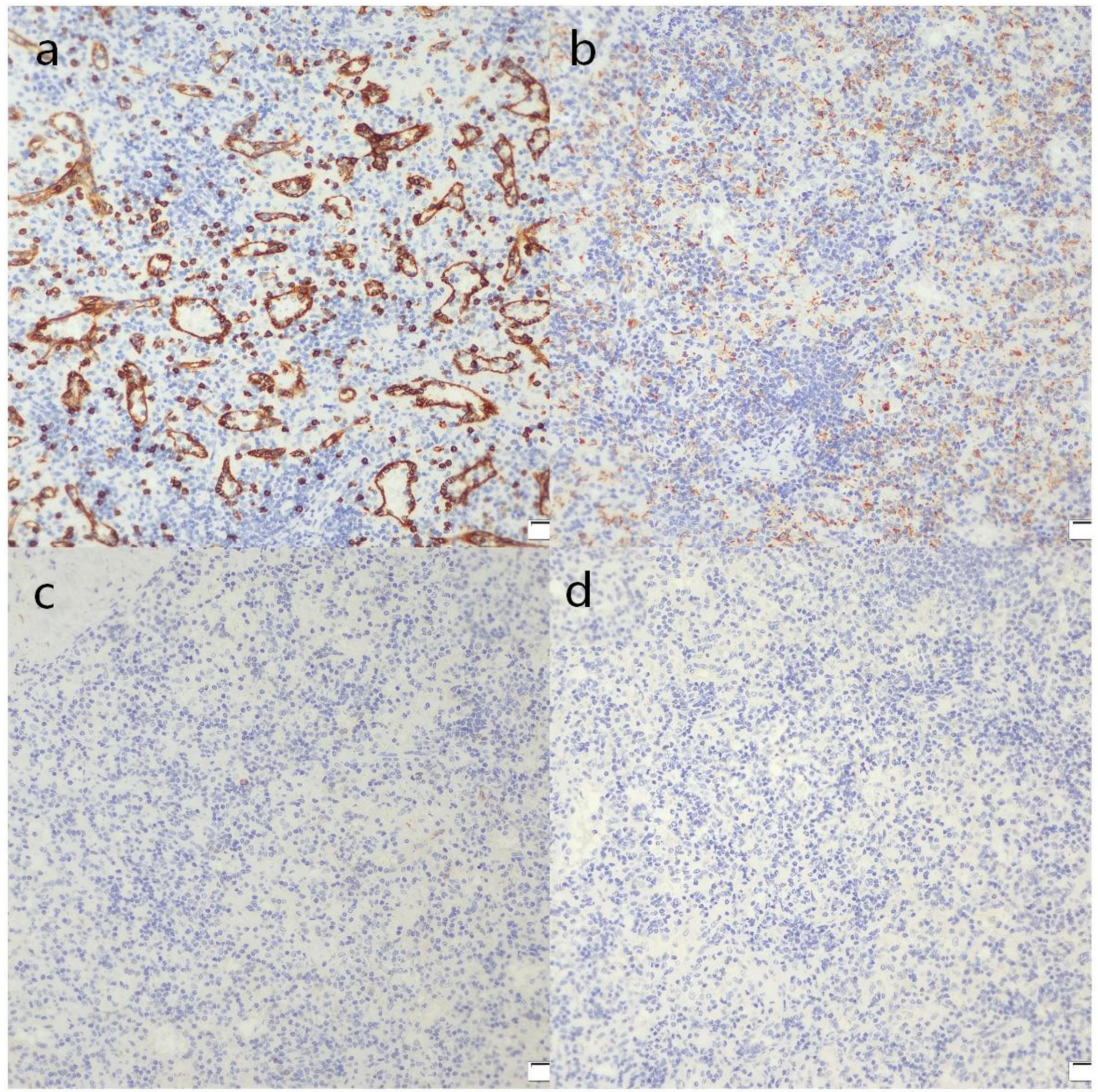
**(a)** Immunohistochemical staining for CD8 highlights sinusoidal endothelial cells, confirming splenic origin (scale bar = 100 μm). **(b)** CD68 staining demonstrates macrophages within the red pulp (scale bar = 100 μm). **(c)** Negative CD117 (c-kit) staining rules out gastrointestinal stromal tumor (GIST) (scale bar = 100 μm). **(d)** Negative DOG1 staining further excludes the possibility of GIST (scale bar = 100 μm).

Definitive diagnosis in this case was established through histopathological examination of the resected specimen. Hematoxylin and eosin (H&E) staining revealed typical splenic architecture, including red pulp, white pulp, and trabeculae, with no evidence of cytologic atypia or malignancy (**Figure 5**). Immunohistochemical staining confirmed the splenic origin: CD8 positivity highlighted the sinusoidal endothelial cells of the red pulp (**Figure 6a**), and CD68 positivity demonstrated the presence of macrophages within the red pulp (**Figure 6b**). Crucially, staining for CD117 (c-kit) was negative, effectively ruling out a gastrointestinal stromal tumor (GIST) (**Figure 6c**). In addition, DOG1 staining was also negative, further excluding the possibility of GIST (**Figure 6d**). This comprehensive immunohistochemical panel provided unequivocal evidence for the diagnosis of splenosis while excluding the primary malignant considerations.

It is essential to differentiate splenosis from pelvic malignancies such as gastrointestinal stromal tumors, metastatic carcinoma, and lymphoma, since misdiagnosis may result in unnecessary overtreatment (6). To facilitate this differentiation, we summarize the key distinguishing features in **Table 1**.

**Table 1:** Key differential diagnostic features between splenosis and malignant pelvic masses (GIST, peritoneal carcinomatosis, lymphoma).

Feature	Splenosis	GIST	Peritoneal Carcinomatosis	Lymphoma
Clinical History	Splenic trauma/splenectomy	Often none	Primary cancer history	B symptoms possible
Imaging (CEUS)	Homogeneous enhancement, persistent	Hypervascular, variable necrosis	Irregular peritoneal thickening, nodular	Homogeneous or heterogeneous
Tumor Markers	Normal	Usually normal	Often elevated (e.g., CA125, CEA)	LDH may be elevated
IHC Profile	CD8+, CD68+; CD117-, Pan-CK-	CD117+ (c-kit), DOG1+	Pan-CK+; organ-specific markers	LCA+, CD20+ or CD3+

The immunohistochemical markers in bold represent the most diagnostically significant antibodies for differential diagnosis. CD8 positivity is pathognomonic for splenic tissue, while CD117 and DOG1 co-expression is characteristic of GIST. Peritoneal carcinomatosis demonstrates epithelial markers consistent with the primary tumor origin. Lymphoma is distinguished by leukocyte common antigen positivity and lineage-specific markers (CD20 for B-cell, CD3 for T-cell phenotypes).

In this case, although contrast-enhanced ultrasound suggested a possible intestinal origin, the absence of gastrointestinal symptoms, negative tumor markers, and lack of definitive evidence of a primary intestinal wall lesion on cross-sectional imaging (CT and MRI) made the evidence for a primary gastrointestinal malignancy inconclusive during multidisciplinary discussions. In retrospect, adhering more strictly to the diagnostic algorithm for suspected gastrointestinal tumors, which prioritizes endoscopic evaluation, would have been valuable. This experience underscores that even when imaging findings are atypical, endoscopic assessment should be proactively considered to definitively rule out mucosal or submucosal primary lesions before proceeding to more invasive diagnostic procedures.

Furthermore, the role of advanced functional imaging, such as ^18^F-fluorodeoxyglucose positron emission tomography/computed tomography (^18^F-FDG PET/CT), in this context warrants discussion. While tumor markers were normal in our patient, it is acknowledged that some malignancies can present without elevated markers and may exhibit variable FDG avidity. In our case, PET/CT was not pursued due to considerations of accessibility, cost, and the potential for diagnostic uncertainty, as both malignant lesions and benign splenic tissue can demonstrate FDG uptake (4). Nevertheless, in complex diagnostic dilemmas where less invasive measures are inconclusive, PET/CT could provide valuable metabolic information to guide management, although histopathological confirmation remains the gold standard.

**Figure 7:**
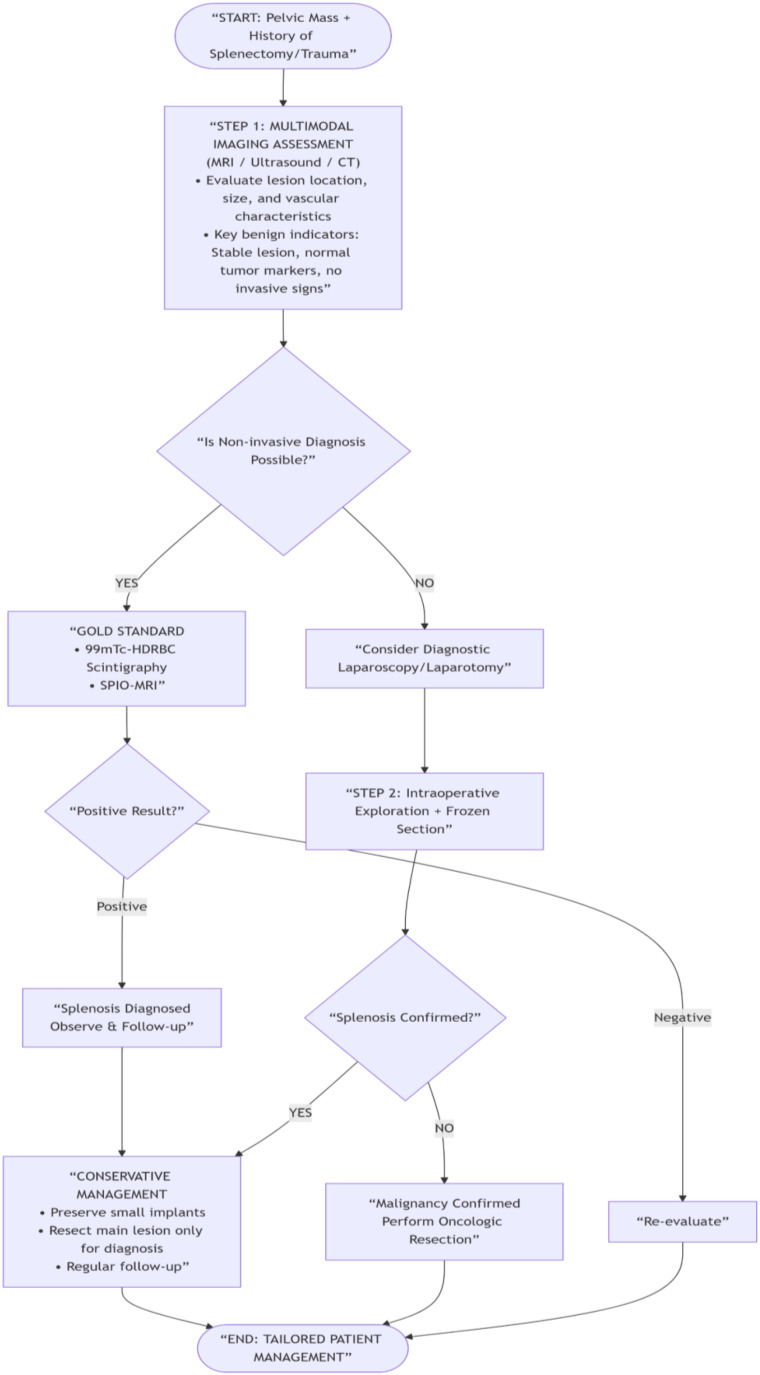
Diagnostic and management algorithm for patients presenting with pelvic masses and a history of splenic trauma or splenectomy.

To minimize the risk of misdiagnosis, close multidisciplinary collaboration among radiologists, surgeons, and pathologists is essential. When imaging findings are inconclusive, radionuclide scintigraphy or biopsy should be considered preoperatively. Based on our experience, we have developed a diagnostic and management algorithm for patients presenting with pelvic masses and a history of splenic trauma or splenectomy (**Figure 7**). This flowchart guides clinicians through the decision-making process, from initial imaging to definitive management, and emphasizes the role of intraoperative frozen section in guiding the extent of resection.

With respect to management, the literature indicates that asymptomatic splenosis does not require surgical intervention, as it carries no malignant potential (12). Resection should be considered only when complications arise, such as torsion, hemorrhage, or obstruction, or when malignancy cannot be excluded (13). In this case, intraoperative frozen section analysis successfully prevented unnecessary extensive resection, underscoring the pivotal role of intraoperative consultation in complex cases. For our patient, who remains asymptomatic, we have recommended annual abdominal ultrasound surveillance, with instructions to return for evaluation if she develops any new symptoms such as abdominal pain or bloating.

Clinically, the case reminds us that splenosis should be considered in the differential diagnosis of pelvic masses, especially in patients with a history of splenic trauma or splenectomy. Greater awareness among clinicians, careful use of imaging, and effective teamwork across specialties are important for reaching an accurate diagnosis and preventing overtreatment.

## 4 Conclusions

Splenosis is a benign condition that is often misdiagnosed as a malignant tumor. Clinicians should maintain a high index of suspicion, especially in patients with a history of splenic trauma or splenectomy. This case yields several key learning points. First, a remote history of splenic trauma or splenectomy is a critical clue that must be actively sought. Second, integrating multimodal imaging findings is essential; MRI may be particularly valuable for assessing lesion stability over time (14). Third, when percutaneous biopsy is technically challenging due to anatomical constraints, diagnostic surgery with intraoperative frozen section is a valid and decisive approach. Fourth, when non-invasive diagnosis (e.g., radionuclide scintigraphy) is unavailable or inconclusive, diagnostic laparoscopy with intraoperative frozen section can provide a definitive diagnosis and guide the extent of resection, preventing overtreatment (15). Fifth, a comprehensive immunohistochemical panel is crucial to confirm splenic origin and exclude malignant mimics. Finally, asymptomatic splenosis can be safely managed with observation, sparing patients unnecessary extensive surgery. Imaging methods such as contrast-enhanced ultrasound and CT often cannot clearly distinguish between benign and malignant lesions. Diagnostic accuracy improves when clinical history, MRI results, radionuclide scans, and input from a multidisciplinary team are considered together. Most reports indicate that asymptomatic splenosis does not need aggressive surgery, and treatment should be tailored to the individual patient. Increasing clinicians’ awareness of this condition and refining diagnostic approaches will help prevent unnecessary operations. Future studies should explore the broader application of functional imaging modalities, such as scintigraphy and SPIO-enhanced MRI, to improve preoperative diagnostic accuracy.

## Data Availability

The original contributions presented in the study are included in the article/supplementary material. Further inquiries can be directed to the corresponding author.
